# Sensory neurons from dorsal root ganglia regulate endothelial cell function in extracellular matrix remodelling

**DOI:** 10.1186/s12964-020-00656-0

**Published:** 2020-10-19

**Authors:** Alice Leroux, Bruno Paiva dos Santos, Jacques Leng, Hugo Oliveira, Joëlle Amédée

**Affiliations:** 1 Univ. Bordeaux, INSERM, BIOTIS, U1026, F-33000 Bordeaux, France; 2 Univ. Bordeaux, CNRS, Solvay, LOF, UMR 5258, F-33006 Pessac, France

**Keywords:** Angiogenesis, Cellular communication, Innervation, Matrix Metalloproteinases, Extracellular matrix Remodelling, Neurovascular interplay

## Abstract

**Background:**

Recent physiological and experimental data highlight the role of the sensory nervous system in bone repair, but its precise role on angiogenesis in a bone regeneration context is still unknown. Our previous work demonstrated that sensory neurons (SNs) induce the osteoblastic differentiation of mesenchymal stem cells, but the influence of SNs on endothelial cells (ECs) was not studied.

**Methods:**

Here, in order to study in vitro the interplay between SNs and ECs, we used microfluidic devices as an indirect co-culture model. Gene expression analysis of angiogenic markers, as well as measurements of metalloproteinases protein levels and enzymatic activity, were performed.

**Results:**

We were able to demonstrate that two sensory neuropeptides, calcitonin gene-related peptide (CGRP) and substance P (SP), were involved in the transcriptional upregulation of angiogenic markers (vascular endothelial growth factor, angiopoietin 1, type 4 collagen, matrix metalloproteinase 2) in ECs. Co-cultures of ECs with SNs also increased the protein level and enzymatic activity of matrix metalloproteinases 2 and 9 (MMP2/MMP9) in ECs.

**Conclusions:**

Our results suggest a role of sensory neurons, and more specifically of CGRP and SP, in the remodelling of endothelial cells extracellular matrix, thus supporting and enhancing the angiogenesis process.

**Video abstract**

**Supplementary information:**

**Supplementary information** accompanies this paper at 10.1186/s12964-020-00656-0.

## Background

Bone is a dynamic tissue which is both vascularized [1–3] and innervated [4–6]. Numerous evidence from animal studies that began in the 1980’s, suggest a relationship between sensory nerve damage and joint diseases [7–9]. The focus on skeletal neurobiology emerged with a series of sensory denervation experimental models performed mainly in rats [10, 11] and confirmed the role of peripheral innervation in the regulation of bone tissue development and regeneration. Nerve fibres exist throughout bone but are more abundant in the periosteum, as well as in the mineralized and vascularized parts of bone tissue, and are predominantly located at metabolically active regions of bone [12–18]. More interestingly, nerves are closely associated with arteries and capillaries. Nerve fibres penetrate through the porous structures of the cancellous bone along with blood vessels, suggesting that they are closely linked to each other molecularly, functionally and anatomically [19, 20]. Important studies brought the attention to the effect of sensory innervation on osteogenesis and angiogenesis. A work published by Fukuda and colleagues revealed that the sensory nervous system (SNS) is the main regulator of bone formation and remodelling in mice. In this work, the authors identified the Semaphorin 3A (Sema3A) produced by sensory neurons as a crucial protein for proper bone mass accrual [21]. More recently, Tomlinson and colleagues demonstrated that sensory nerves coordinate the vascularization and ossification of developing endochondral bone and that, when blocking specific sensory innervation and inhibiting specific functional sensory signalling, vascular invasion of the primary ossification centre was strongly impaired [22]. Thus, the understanding of the neuro-vascular interplay, in a bone regeneration context, will open new avenues on therapeutic approaches that can sustain/modulate angiogenesis, a key process in bone remodelling and repair.

Indeed, both systems share common molecules to maintain homeostasis and function [23–27]. Such examples are platelet-derived growth factor and vascular endothelial growth factor (VEGF) that, besides being key molecules for angiogenesis, also enhance the survival, proliferation and migration of glial cells and stimulate the axonal outgrowth [28, 29]. In addition, it has become apparent that endothelial tip cells also respond to neural guidance cues, including Netrins, Slits, Semaphorins and Ephrins [30, 31], providing a possible cellular interplay between both cell types in order to form the complex nerve/vessel network during tissue repair. Moreover, it has been shown that sensory neurons, associated with Schwann cells, are able to secrete VEGF and are responsible for the patterning of arterial differentiation and blood vessel branching in the skin [32], where a close relation between innervation and vascularization is found.

Moreover, previous studies have shown that during bone repair, innervation precedes angiogenesis [33], leading to the hypothesis that the peripheral innervation may be essential for neovascularization and then for bone regeneration, by the local release of neuronal mediators. Apart from these common guidance molecules, among the sensory neuropeptides identified in bone, substance P (SP), a mediator of nociception and inflammation, and calcitonin gene-related peptide (CGRP), a potent peptide vasodilator and mediator in the transmission of pain with consistent osteo-anabolic effect, can also contribute to angiogenesis [34–36]. The receptor for substance P, neurokinin 1 (NK1), is found to be expressed by endothelial cells (ECs) and the in vitro exposure of ECs to SP has shown to induce a concentration-dependent migration of these vascular cells [37]. The receptors for CGRP have also been described in ECs [38–40] and CGRP has shown to promote proliferation and inhibit apoptosis in ECs [41]. In a rat knee-joint model, intra-articular CGRP injection increased ECs proliferation, while inhibition of calcitonin receptor-like receptor (CRLR) - RAMP 1 attenuated ECs proliferation in capsaicin-induced knee joint synovitis [42].

The effect of innervation and neuropeptides is also described in cutaneous wound healing. A significantly higher number of skin nerve fibres were observed in normotrophic scars compared with hypertrophic scars after burn, suggesting a regulatory role for the skin nerve system in the outcome of burn wound healing [43]. The direct neuronal contact in vitro accelerates the fibroblast differentiation into myofibroblasts, which secrete collagen fibres and induce wound contraction [44]. Finally, neuropeptides such as SP and CGRP seem to modulate the expression of collagens during skin wound healing [45].

Although challenging, more biological studies are needed to investigate the control of neural signals on ECs function and activity, by using relevant in vitro models that mimic the innervation process of a vascularized extracellular matrix. In our previous work, by using microfluidic devices for indirect co-cultures assays, we have demonstrated that in vitro, dorsal root ganglia (DRGs) neurons isolated in rats were able to enhance the osteogenic differentiation of rat mesenchymal stromal cells (MSCs) [46]. These microfluidic chambers were designed to mimic the innervation process, where cell interactions are made through axons emitted by neurons toward peripheral tissues. These devices also permitted to analyse separately, at proteomic and transcriptomic levels, the two cell populations and thereafter evaluate the influence of sensory neurons during the osteogenic differentiation of mesenchymal stem cells.

Here, we designed a new platform of devices for co-culturing sensory neurons in a central compartment, and rat bone marrow-derived endothelial cells in two lateral compartments. In this work we demonstrate that sensory neurons are able to modulate the transcriptional and translational profiles of endothelial cells towards extracellular matrix (ECM) remodelling, involving activities of extracellular Matrix MetalloProteinases (MMPs), such as MMP2 in particular. By using specific antagonists, we also show that the two neuropeptides CGRP and SP are involved in this process.

## Methods

### Microfluidic devices fabrication

Microfluidic devices moulds were obtained using standard photolithography and soft lithography procedures [47]. Dimension measurements of the moulds were performed through interferometry using an S neox 3D Optical Profiler confocal microscope (Sensofar® Metrology). The devices were made with polydimethylsiloxane (PDMS) (Sylgard® 184, Sigma-Aldrich cat. n° 761,036) and polymerized at 60 °C for 3 h. Before use, devices were incubated for 4 days in absolute ethanol, dried and then exposed to UV light (254 nm) for 40 min.

### Cell isolation and culture

Primary sensory neurons (SNs) were obtained from dorsal root ganglia (DRGs) from healthy 5–8 weeks old female Wistar rats, as described by Malin and colleagues [48]. Briefly, spinal columns were removed and opened from the caudal to the rostral region in order to expose the DRGs. The DRGs were individually harvested and digested with 2800 U/mL of Collagenase Type IV (Gibco®) for 1 h at 37 °C. Subsequently, digestion products were washed twice with Dulbecco’s Modified Eagle’s Medium 1 g/L glucose (DMEM, Gibco®) and mechanically dissociated using fire-polished glass Pasteur pipettes (full diameter and ½ diameter). The cell suspension was finally washed three times with culture medium, and resuspended in DMEM 4.5 g/L glucose supplemented with 2% (v/v) B-27 Serum-Free Supplement® (B-27, Gibco®), 1% (v/v) fetal bovine serum (FBS, PAN™ Biotech, Aidenbach, Germany) and 1% (v/v) Penicillin Streptomycin (Pen/Strep, Gibco®). Bone marrow-derived endothelial cells (ECs) were purchased from Cell Biologics® (catalog number RA-6221). ECs were cultured with endothelial cell growth medium-2MV (EGM-2MV; Lonza-Verviers, France) and incubated at 37 °C in humidified atmosphere with 5% CO_2_. Cells were used between passages 5 to 8.

Calcitonin gene related peptide (CGRP) and substance P (SP) (Interchim, catalog numbers RP1109560.5 and RP10178 respectively) were used directly in culture medium of ECs cultivated in 48-well plates, in concentrations varying from 0.1 nM to 100 nM for CGRP [41] and from 10 nM to 10 μM for SP [37]. Cells were seeded at 10^4^ cells/cm^2^.

For co-culture assays, microfluidic devices were electrostatically attached over glass coverslips previously coated with 0.1 mg/mL Poly-D-Lysine (PDL, Sigma-Aldrich) and 20 μg/mL laminin (Sigma-Aldrich). ECs were seeded in the lateral compartments at a density of 80 cells/mm^2^. SNs were seeded in the central compartment immediately after isolation at a density of 160 cells/mm^2^. Fresh medium was added every 2 days and the cells were maintained in culture until day 7.

The antagonists BIBN4096 (for CGRP) and SR140333 (for SP) (Tocris, Bio-Techne) were added directly in ECs culture medium at a concentration of 10 μM each [49–51].

### Immunofluorescence staining

SNs and ECs were fixed with 2% (w/v) paraformaldehyde for 30 min at room temperature (RT), permeabilised with 0.1% (v/v) Triton X-100 for 5 min at RT and blocked with 1% (w/v) bovine serum albumin (BSA, GE Healthcare) for 30 min at RT. For SNs, primary rabbit anti-rat β-III tubulin antibody (Abcam n°18207) was used at a 1:500 dilution at 4 °C overnight. Secondary goat anti-rabbit IgG conjugated with Alexa Fluor 488 (Invitrogen™ n°R37116) was used at a 1:400 dilution for 45 min at RT. Filamentous actin of ECs was labelled with rhodamin-phalloidin (Invitrogen™ n°R415) diluted at 1:400. Nuclei were labelled with DAPI (4′, 6′-diamidino-2-phenylindole, Life Technologies™) at 1 μg/mL for 5 min at room temperature. Images were acquired in a Leica TCS SPE 5 Confocal Laser Scanning Microscope.

### Transmission Electron microscopy (TEM)

SNs and ECs co-cultivated in the microfluidic devices were fixed with 2% (v/v) paraformaldehyde, 2% (v/v) glutaraldehyde in 0.1 M sodium cacodylate buffer (pH 7.4) during 1 h at RT and washed in 0.1 M sodium cacodylate buffer (pH 7.4). They were then post-fixed in a mix of 1% osmium tetroxide (v/v) / 1% potassium ferricyanide K3Fe (CN)6 (w/v) in 0.1 M sodium cacodylate buffer during 2 h, on ice and in the dark. After washing in water, samples were stained in block in 0.5% (w/v) aqueous uranyl acetate solution during 30 min, in the dark, at room temperature. Subsequently, cells were washed in water then dehydrated through a series of graded ethanol and embedded in a mixture of pure ethanol and epoxy resin (Epon 812; Delta Microscopies, Toulouse, France) 50/50 (v/v) during 2 h and then in 100% resin overnight at RT. The polymerization of the resin was carried out over a period between 24 and 48 h at 60 °C. Samples were then sectioned using a diamond knife (Diatome, Biel-Bienne, Switzerland) on an ultramicrotome (EM UCT, Leica Microsystems, Vienna, Austria). Ultrathin sections (65 nm) were picked up on slot copper grids with formvar membrane and then stained with Uranyless (Delta Microscopies, Toulouse, France) and lead citrate. Grids were examined with a Transmission Electron Microscope (H7650, Hitachi, Tokyo, Japan) at 80 kV, equipped with a camera Orius 11 Mpixel (Roper Scientific, France).

### RNA extraction, cDNA synthesis and RT-qPCR analysis

Total RNA was extracted from ECs using the RNeasy Plus Micro Kit (Qiagen) according to the manufacturer’s protocol. RNA concentration and purity (OD 260/280) were determined using a NanoPhotometer P330 (Implen GmbH). 100-500 ng of total RNA were reverse transcribed into cDNA using the Maxima Reverse Transcriptase kit (Thermo Scientific™, Thermo Fisher Scientific, Waltham, MA, USA), according to the manufacturer’s protocol. RT-qPCR experiments were performed using a Takyon™ No Rox SYBR® 2X MasterMix dTTP blue (Kaneka Eurogentec S.A., Belgium) with standard PCR conditions for Sybr Green® detection in a CFX Connect™ Real-Time PCR Detection System (Bio-Rad Laboratories, Hercules, CA, USA) and analysed with the CFX Manager™ software, version 3.0 (Bio-Rad Laboratories). Primer sequences are presented in Table 1. Target gene expression was quantified using the cycle threshold (Ct) values and relative mRNA expression levels were calculated according to the 2^-DDCt^ method [52]. Ribosomal protein lateral stalk subunit P0 (*Rplp0*), glyceraldehyde 3-phosphate dehydrogenase (*Gapdh*) and hypoxanthine phosphoribosyl transferase 1 (*Hprt1*) were used as reference genes.

**Table 1:** Sequences of primers used to analyze ECs gene expression

Gene	Gene ID	Sequence	Fragment length (bp)
*Angpt1*	89807	F: 5′ GCTAACAGGAGGTTGGTGGT 3′ R: 5′ CACTTTATCCCATTCAGTTTTCCA 3’	102
*Col4a1*	290905	F: 5’ GAACGAAAGGGACACGAGGAC 3′ R: 5′ CAAGCCAGGAGGACCGAGTG 3’	158
*Pecam1*	29583	F: 5’ CCCTGTCCCCACCCAAAG 3′ R: 5′ ACCTCCTCTCACCTCCCAAA 3’	173
*Vegfa*	83785	F: 5’ CTTCTTCCACCACTGTGTCT 3′ R: 5′ GCCTCAGGACATGGCACTAT 3’	200
*Mmp2*	81686	F: 5’ CCCTCCCCTGATGCTGATAC 3′ R: 5′ CTGTCCGCCAAATAAACCGAT 3’	157
*Rplp0*	64205	F: 5’ CACTGGCTGAAAAGGTCAAGG 3′ R: 5′ GTGTGAGGGGCTTAGTCGAA 3’	187
*Gapdh*	24383	F: 5’ GGCATTGCTCTCAATGACAA 3′ R: 5′ TGTGAGGGAGATGCTCAGTG 3’	213
*Hprt1*	24465	F: 5’ AGCCTAAAAGACAGCGGCAA 3′ R: 5′ GGCCACAGGACTAGAACGTC 3’	87

### MMP2/MMP9 concentration

After 4 and 7 days of co-culture, ECs culture medium was harvested and the cells were lysed in PBS 0.01 M containing 1% (v/v) Triton X-100 (Merck) and protease inhibitor cocktail (Sigma-Aldrich). Mmp2/Mmp9 concentration was measured by InnoZyme™ Gelatinase (MMP2-MMP9) activity assay kit (Cat. No. CBA003 Calbiochem) according to the manufacturer’s protocol. Briefly, gelatinase control for the standard curve and samples were diluted in activation buffer and distributed in the 96-wells plate, substrate working solution was added to each well and the plate was incubated at 37 °C for 6 h. The fluorescence was measured using a FLUOstar OPTIMA (BMG Labtech) plate reader at an excitation wavelength of 340 nm and an emission wavelength of 405 nm. MMP2/MMP9 concentration was normalized to total protein concentration measured in cell lysates with the Pierce™ BCA (BiCinchoninic Acid) protein assay kit (Thermo Scientific) according to the manufacturer’s protocol.

### Matrix Metalloproteinases activity (Zymography)

The culture supernatants from ECs were harvested after 4 and 7 days of culture in presence or absence of SNs for MMP2/MMP9 activity analysis through zymography. For each sample, 70 ng of proteins were loaded into the wells with a loading buffer of 10% (w/v) SDS, 20% (v/v) glycerol, 0.1% (w/v) bromophenol blue and 1 M Tris-HCl (pH 6.8). The acrylamide/bis-acrylamide (30% / 0.8% w/v) gel was composed of a running gel (pH 8.8) with 2% (w/v) gelatin and a stacking gel (pH 6.8). After 3 h of electrophoresis at 100 V, the gel was washed four times in a washing buffer containing 2.5% (v/v) Triton X-100. The gel was then transferred in a renaturation buffer with 50 mM Tris-HCl (pH 7.4), 50 mM NaCl and 10 mM CaCl_2._ After 20 h of incubation at 37 °C under agitation, the gel was incubated in a staining solution of 0.5% (w/v) Coomassie Brilliant Blue, 50% (v/v) ethanol and 10% (v/v) acetic acid for 90 min. The gel was then destained in a solution containing 50% (v/v) absolute ethanol and 10% (v/v) glacial acetic acid at room temperature overnight. The white bands area was quantified for each sample using ImageJ software [53].

### Statistical analysis

Data is expressed in mean values ± standard deviation. Differences between groups were analysed with the software Prism 5.0 (GraphPad Software Inc.) by Student’s *t* test or one-way ANOVA followed by Dunnet post hoc test when necessary. A value of *p* < 0.05 is considered statistically significant.

## Results

### SNs emit neurites towards ECs, closely interacting

**Fig. 1:**
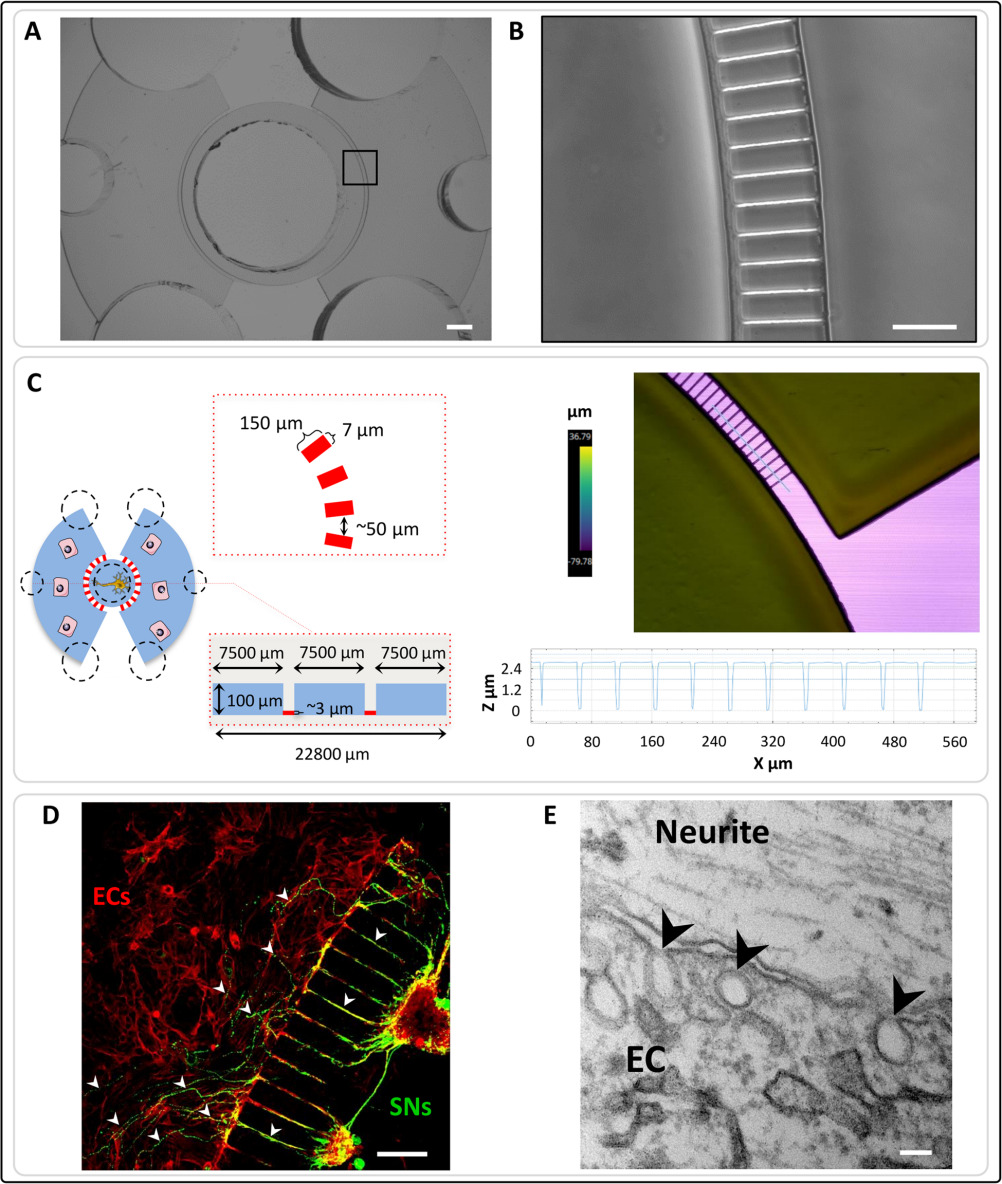
Microfluidic devices validation. **a** – **b** Conventional photolithography techniques were used to create the microfluidic devices with precise and specific dimensions. Scale bars = 100 μM. **c** Scheme of the microfluidic devices dimensions (left). Profilometry was performed to confirm the microchannels dimensions (right). **d** Sensory neurons (SNs) derived from rat dorsal root ganglia were seeded in the central compartment of the devices. Rat bone marrow derived endothelial cells (ECs) were cultivated in the lateral compartments. After 4 days of culture, cells were fixed and stained with β-III tubulin (SNs in green) and phalloidin (ECs in red). The neurites emitted by the sensory neurons were able to cross the microchannels and go from the central compartment to the lateral ones (white arrowheads) where they closely interact with endothelial cells. Scale bar = 100 μm. **e** Transmission Electron Microscopy was performed to confirm this close interaction and showed vesicles in ECs (black arrowheads). Scale bar = 100 nm

In order to analyse the impact of SNs on ECs function, a new microfluidic device was engineered for co-culturing both cell types (Fig. 1a-b). This device is composed of one central compartment for SNs cell culture and two lateral compartments for ECs. The microchannel are 150 μm long and their height (2.73 ± 0.03 μm) was checked using profilometry techniques (Fig. 1c).

After 4 days of co-culture, SNs emit neurites through the microchannels towards ECs (Fig. 1d), indicating that this new design of microfluidic devices proposed by our group is functional. Transmission electron microscopy confirmed a close interaction between ECs and neurites. Tight junctional contact between cell membranes was not observed but numerous vesicles were seen inside ECs in the vicinity of cell membranes (Fig. 1e).

### Specific genes for EC function are regulated by SNs

**Fig. 2:**
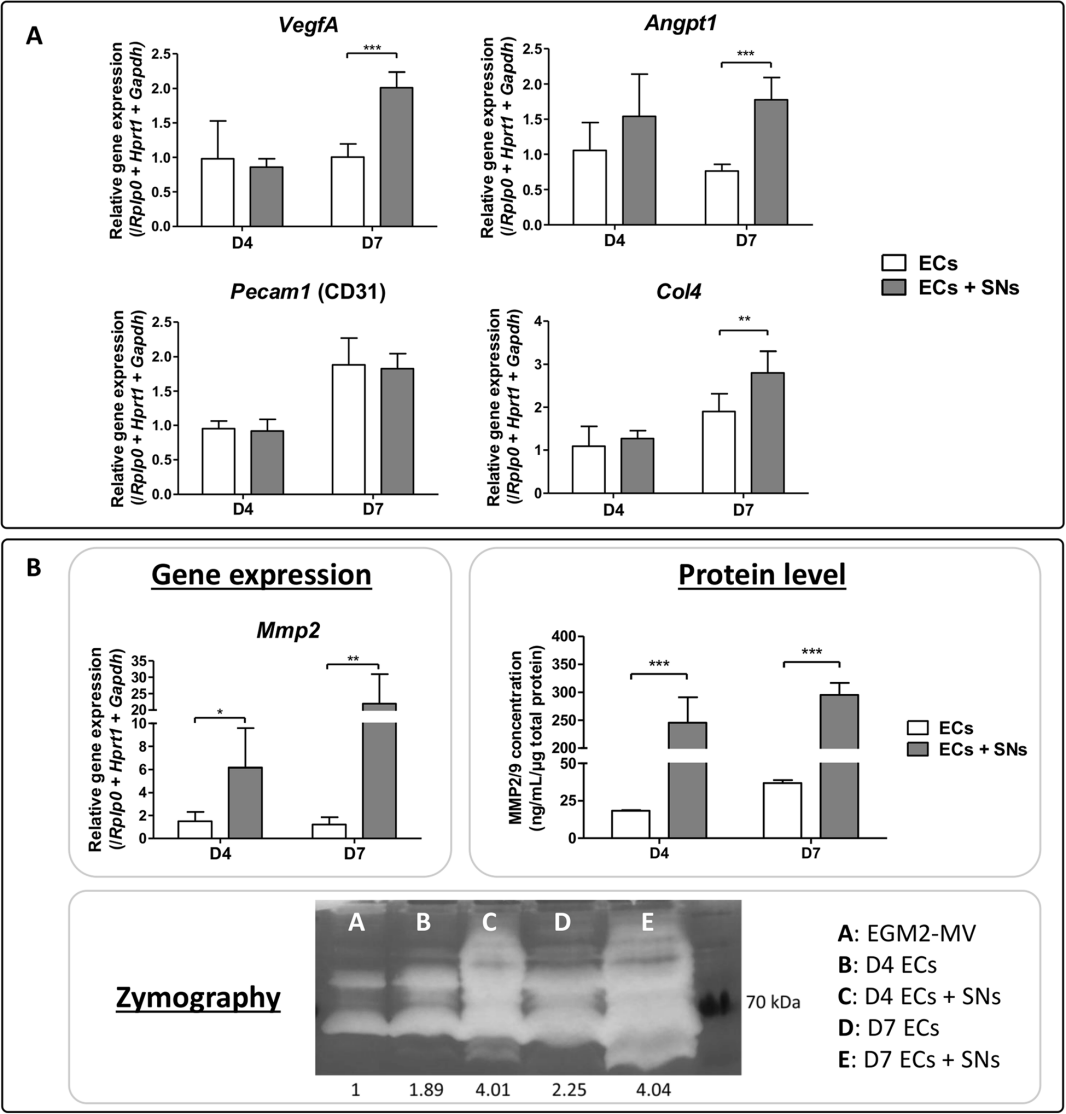
Transcriptional and translational regulation of ECs by SNs. **a** Gene expression analysis of angiogenic markers expressed by endothelial cells cultivated in the microfluidic devices in presence (grey bars) or absence (white bars) of sensory neurons in the central compartment. After 4 and 7 days of culture, cells were harvested, RNA was extracted and RT-qPCR was performed. Gene expression of *Angpt1*, *VegfA* and *Col4* is upregulated in ECs when co-cultivated with sensory neurons after 7 days of culture. The graphs represent mean values ± SD (*n* = 4 independent experiments, unpaired t test, ** *p* < 0.01, *** *p* < 0.001). **b** *Mmp2* gene expression is upregulated at both time points in presence of sensory neurons (*n* = 4 independent experiments, unpaired t test, * *p* < 0.05, ** *p* < 0.01). Concentration of MMP2 and MMP9 was measured in ECs’ supernatants cultivated with (grey bars) or without (white bars) sensory neurons. At both time points, MMP2/MMP9 concentration is increased in ECs when they are co-cultivated in presence of SNs (*n* = 5 microfluidic devices, unpaired t test, *** *p* < 0.001). Representative picture of enzymatic activity of matrix metalloproteinases measured by zymography. The band intensity was quantified using ImageJ software. In presence of sensory neurons, ECs show an increase of MMP2/MMP9 enzymatic activity at day 4 and day 7 (pool of *n* = 6 microfluidic devices)

In order to study the influence of SNs on ECs function, the transcriptional expression of some specific genes was analysed in ECs cultured in the microfluidic devices, in presence or absence of SNs. At day 7, in the presence of SNs, ECs show an upregulation of *Angpt1* (1.7-fold), *VegfA* (2-fold) and *Col4* (2.8-fold) relative to ECs in monoculture (Fig. 2a). No difference in gene expression was observed for *Pecam1* after 4 and 7 days of culture (Fig. 2a). More interestingly, an upregulation of *Mmp2* gene expression was observed at day 4 (6-fold) and day 7 (22-fold) when ECs were co-cultured with SNs (Fig. 2b).

### SNs increase matrix metalloproteinases production and activity by ECs

To further analyse if SNs could induce the production of matrix metalloproteinases, the protein concentration of MMP2/MMP9 was measured in the supernatant of ECs cultivated with and without SNs (Fig. 2b). A significant increase of MMP2/MMP9 concentration was observed when ECs were co-cultured with SNs after 4 (13-fold) and 7 days (8-fold) of culture, relative to ECs alone at the same time point. This confirms our previous results regarding *Mmp2* upregulation at the gene expression level (Fig. 2b). When the metalloproteinases activity was measured by zymography, the same tendency was observed. The semi-quantification of bands revealed increased enzymatic activity when ECs were co-cultured with SNs. In addition, bands with different molecular weights are observed, suggesting that different isoforms of MMP2/MMP9 are expressed when ECs are co-cultured with SNs (Fig. 2b).

### CGRP and SP molecules modulate ECs gene expression

**Fig. 3:**
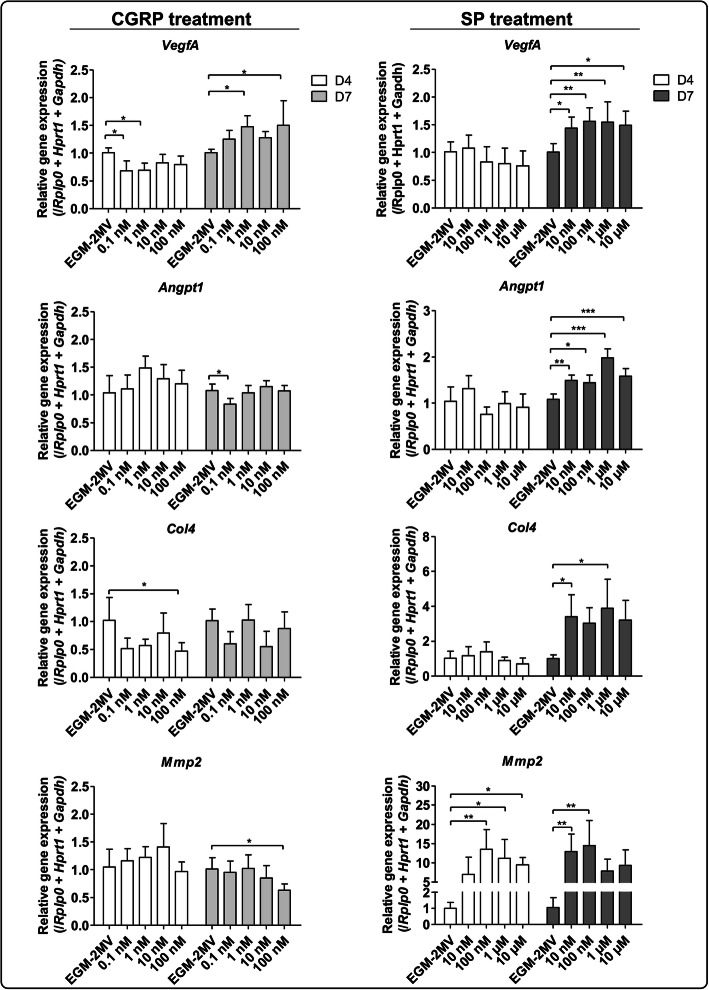
Effect of CGRP and SP on ECs transcriptional profiles. ECs cultivated in 48-wells plates were treated either with CGRP (from 0,1 nM to 100 nM) or SP (from 10 nM to 10 μM) for 4 days (white bars) or 7 days (grey bars) of culture. Total RNA was extracted followed by *Vegfa*, *Angpt1*, *Col4*, and *Mmp2* gene expression analysis by RT-qPCR. *VegfA* expression is upregulated by CGRP after 7 days of culture whereas *VegfA*, *Angpt1*, *Col4* and *Mmp2* expression is upregulated by SP. (*n* = 3 independent experiments, One-way ANOVA followed by post hoc Dunnett’s test, * *p* < 0.05, ** *p* < 0.01, *** *p* < 0.001)

The extension of growing axons depends on extracellular guidance molecules, surface receptors, and signal transduction pathways activated in both cell types [20, 54]. Calcitonin gene-related peptide (CGRP) and substance P (SP) are two important neuropeptides synthesised by a subpopulation of peptidergic nociceptors SNs [55]. They are known to have roles in nociception [56, 57], and can modulate bone cells and ECs migration and proliferation [41, 51]. In order to investigate their potential role in the effects previously described in the sections above, we first supplemented the ECs medium with different concentrations of CGRP and SP (Fig. 3).

In the presence of CGRP, *Vegfa* was downregulated at day 4 with 0.1 nM and 1 nM compared to cells cultured in EGM-2MV (Fig. 3), but this downregulation happened only during this early time point. Conversely, at day 7, its expression was upregulated at 1 nM and 100 nM relative to EGM-2MV alone (Fig. 3). No differences were observed in *VegfA* expression at day 4 when ECs were treated with different concentrations of SP. However, after 7 days of culture, a slight but significant upregulation was observed for each SP concentration (Fig. 3). In the presence of CGRP at day 4, no differences were observed in the expression of *Angpt1*, but a slight downregulation was observed at day 7 with 0,1 nM of CGRP. For the SP treatment, *Angpt1* was significantly upregulated at day 7 for each concentration. Similarly to *VegfA*, there was also a downregulation of *Col4* at 100 nM of CGRP relative to EGM-2MV. This slight downregulation only happened at an early time point, but here no significant differences were observed at day 7. However, *Col4* was significantly upregulated when treated with 10 nM of SP compared to EGM-2MV. For *Mmp2*, only a slight downregulation of the gene expression was detected after 7 day of culture with 100 nM of CGRP. Nevertheless, with the SP treatment, *Mmp2* was upregulated at both time points. At day 4, there was a clear upregulation with concentrations of 100 nM, 1 μM and 10 μM of SP, and at day 7, with 10 nM and 100 nM. Importantly, the peak of upregulation was around 13-fold at both time points with 100 nM of SP (*p* < 0.01).

**Fig. 4:**
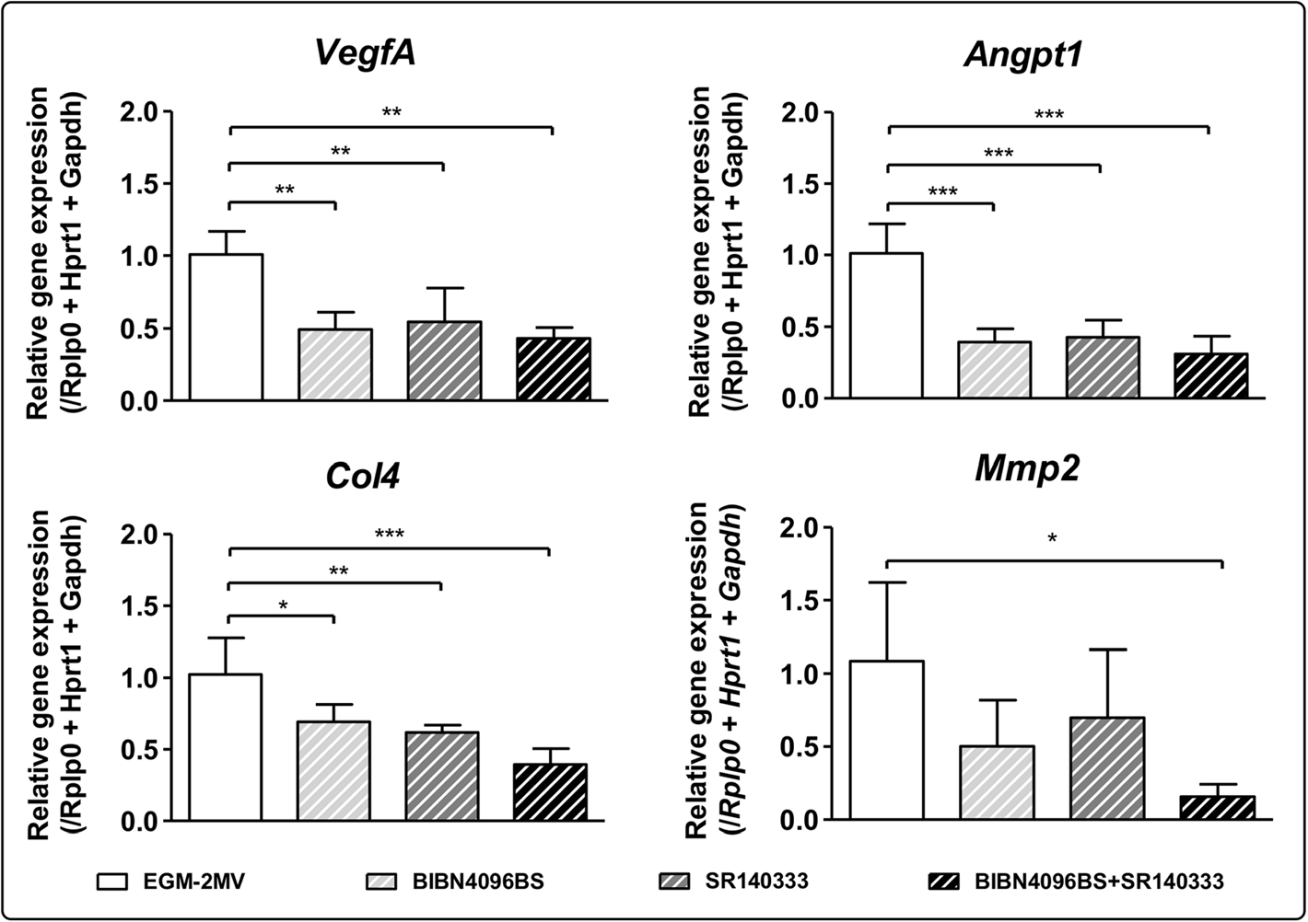
Effect of the inhibition of CGRP and SP on ECs transcriptional profiles. ECs were cultivated for 7 days in the microfluidic devices in presence of sensory neurons. Endothelial cells were either not treated (EGM-2MV) or treated with CGRP antagonist (BIBN4096BS), SP antagonist (SR140333) or both, at a concentration of 10 μM each. Total RNA was extracted and gene expression of *VegfA*, *Angpt1*, *Col4* and *Mmp2* was analyzed by RT-qPCR. CGRP and SP antagonists downregulate ECs gene expression of *Angpt1*, *VegfA* and *Col4*, both separately and together. *Mmp2* gene expression is only significantly downregulated when ECs were treated with both antagonists. (n = 6 microfluidic devices, One-way ANOVA followed by post hoc Dunnett’s test, * *p* < 0.05, ** *p* < 0.01, *** *p* < 0.001)

In order to confirm the distinct roles of CGRP and SP in the modulation of ECs function, ECs were co-cultured in microfluidic devices with SNs, in the presence of CGRP and SP antagonists (BIBN4096 and SR140333, respectively) (Fig. 4). After 7 days of culture, *VegfA*, *Angpt1* and *Col4* expressions were downregulated in the presence of CGRP and SP antagonists used either separately or combined. Moreover, even if *Angpt1* and *Col4* expressions were upregulated only in the presence of SP (Fig. 3), we observed not only a downregulation of gene expression when SP is inhibited by its antagonist, but also a downregulation in the presence of CGRP antagonist alone and combined with SP antagonist (Fig. 4).

In addition, there was a slight but not significant downregulation of *Mmp2* in the presence of CGRP or SP antagonists used separately. However, when both antagonists were combined, *Mmp2* was significantly downregulated (6.75-folds).

## Discussion

The neurovascular guidance has been largely described in the literature. Recent studies of embryonic development using time-lapse imaging techniques with transgenic mice and fish, for example, revealed a unique pattern of cellular behaviour, in which cells move along blood vessels. Neurovascular guidance has been reported for endothelial cells that form the lymphatic vascular system in the zebrafish trunk [58] and for oligodendrocyte precursor cells (OPCs) in mice [59], but the molecular mechanisms regulating the cellular communication of SNs with ECs remain largely unknown. Although challenging, more biological studies are needed to investigate the control of neural signals on ECs function and activity, by using relevant in vitro models that mimic the innervation process of a vascularized extracellular matrix.

In the present study we created a functional microfluidic device design to study the interaction between neurons and ECs. Confocal microscopy confirmed that SNs emit neurites towards ECs (Fig. 1d) and TEM suggested that cellular communication may be mediated by soluble factors, due to the absence of direct contact between cell membranes and the presence of several vesicles enclosed in ECs. Regarding the co-culture models, most established in vitro neuro-vascular models use direct co-culture models or transwell-like approaches to investigate either paracrine or juxtacrine cross-talk between the two cells types [60]. Here, the aim of using these devices was to better mimic the physiological microenvironment between nerves and peripheral tissue, and in particular between SNs and ECs (Fig. 1e). Indeed, by cultivating SNs in the central compartment and ECs in the lateral ones, the cellular bodies of both cell types remain separated and interact through the neurites emitted by neurons. Moreover, each cell type can be cultivated in its specific culture medium and each cell population can be analysed separately. Furthermore, having two compartments for ECs allows us to obtain more cellular material for further analysis. A similar approach was described by Osaki et al. [61], but in this work the microfluidic platform was used to co-cultivate in direct contact motor neurons and endothelial cells. In our system, we have focused instead on the study of the impact of sensory innervation. In addition, our in vitro co-culture models were totally deprived of exogenous neurotrophic growth factors, such as NGF [62], and the neurite outgrowth towards the ECs compartment only depended on guidance molecules and paracrine growth factors produced by ECs. It is already described that ECs secrete BDNF that significantly stimulates neurite outgrowth [20] and even BDNF-based therapies are considered an attractive possibility for the prevention/treatment of various brain diseases [63].

Other interesting models to study in vitro innervation were created for the skin, at cellular and tissue level. At cellular level, Château Y et al. evaluated neuro-epidermis connections through a tri-compartmentalized co-culture model between epidermal cells, DRG neurons, and spinal cord cells during 15 days [64]. DRG neurons formed neurites, which connected the neurons compartment with the other cell types. The authors demonstrated the functionality of this model by heat or pain stimulation on epidermal cells compartment and assessing electrophysiology response in DRG neurons. At tissue level, Muller et al. developed an entirely human functional innervated skin model to study cutaneous neuroinflammation [65]. They engineered epidermis and dermis using collagen sponges seeded with keratinocytes, endothelial cells and fibroblasts, and used both SNs and Schwann cells derived from induced pluripotent stem cells (iPSCs) to promote innervation. The iPSC-derived SNs could only emit neurites through dermis and reach epidermis, localized on the top of the tissue culture, in the presence of iPSC-derived Schwann cells and produce sensory neuropeptides in case of direct neuron stimulation.

Here, regarding the impact of the indirect co-culture after 4 and 7 days, markers involved i) in vascular formation and remodelling such as angiopoietin 1 (*Angpt1)*, platelet endothelial cell adhesion molecule 1 (*Pecam1)* vascular endothelial growth factor A (*Vegfa*) and ii) in extracellular matrix remodelling such as type 4 collagen (*Col4*) and matrix metalloproteinase 2 (*Mmp2*) were analysed (Fig. 2). Angiopoietin 1 signalling stimulates migration, proliferation and differentiation of vascular cells [66]. It is closely related to the control of angiogenesis, playing a role in the enlargement of existing vessels, and is essential for vascular maturation and stability [67]. VEGFA is a master growth factor well-known for inducing angiogenesis and endothelial cell growth by promoting cell migration [68]. PECAM1 is a major constituent of endothelial cell intercellular junction and is implicated in several functions, including trans-endothelial migration of leukocytes, angiogenesis, and integrin activation [69]. MMP2 is a matrix metalloproteinase which degrades the extracellular matrix, including type 4 collagen, an important component of the vascularized extracellular matrix [70]. We observed that after 7 days ECs show an upregulation of *Angpt1*, *VegfA* and *Col4* when they are co-cultured with SNs (Fig. 2a), and an upregulation of *Mmp2* at both time points (Fig. 2b). In addition, MMP2/MMP9 protein concentration and enzymatic activity are enhanced at both time points. These results show that SNs play an important role in EC functions by triggering angiogenesis and inducing ECM remodelling, in a temporally controlled way. This is represented by the upregulation of *Mmp2* at day 4 for ECM remodelling, and by *Angpt1*, *Col4* and *VegfA* upregulation at day 7 for ECs migration, and subsequently vessel formation [71–73].

In order to identify the mechanism of action, which modulates ECs functions, we analysed the response of ECs with medium-supplemented SP and CGRP (Fig. 3). They are well-described SNs neuropeptides known to have roles in nociception [56, 57], in the modulation of bone cells and in ECs migration and proliferation [41, 51]. Interestingly, in the treatment with CGRP, a downregulation of *Col4* at 100 nM and *VegfA* at 0.1 nM and 1 nM was observed at day 4, but this effect was not reproduced at day 7, suggesting a downregulation effect only during an early time point. Inhibition and stimulation at cellular/tissular levels are complex phenomena that depend on concentration, lifetime of the signal, cellular/tissular sensitivity and mechanism of inhibition and/or stimulation. According to their concentration, some molecules can have both stimulatory and inhibitory effects on the same cell type. For example, Boeloni and colleagues observed that collagen synthesis in bone marrow mesenchymal stem cells during 21 days of osteogenic differentiation is enhanced when cells are treated with 1 nM - 1 pM of 3, 3′, 5-triiodo-L-thyronine (T3 hormone) but a contrary effect is observed when cells are treated with 100 nM of T3 hormone relative to cells treated in basic medium [74]. For *Mmp2*, only a slight downregulation was observed when ECs were treated with the highest concentration of CGRP at day 7, but an important upregulation was only observed when the culture medium was supplemented with SP, at both time points. Moreover, this upregulation was in the same range as the one observed using a co-culture model. Taken together, these results confirm the potential role of peptidergic nociceptors SNs, through the secretion of SP and CGRP, in the regulation of genes involved in ECs functions. Moreover, there is another indication of the SNs temporal control on ECM remodelling, represented by the upregulation of *Mmp2* at day 4, and on vessel formation, represented by *Angpt1*, *Col4* and *VegfA* upregulation at day 7.

Our results suggest important roles for CGRP and SP: CGRP playing a role in *VegfA* expression modulation and SP playing a major role in *Mmp2* expression modulation. Interestingly, the endogenous production of *VegfA* by ECs is reported to sustain vascular integrity, cellular viability and endothelial homeostasis [75, 76]. It maintains a basal expression level for VEGFR-2 and its downstream signalling activation, controls expression of key endothelial specific genes including TIE-2, and vascular endothelial cadherin through the control of the transcription factor Foxc2 activity. Foxc2 is crucial for ECs migration and microvessel formation in vitro [77] and plays important roles in vascular formation [78]. Moreover, heterozygous mutant (*Foxc2+/−*) mice show significantly decreased expression of *Vegf* and *Mmp2,* and the haplo-deficiency of *Foxc2* results even in impaired formation of tumour blood vessels as well as reduced tumour growth [79].

Some other data are available in the literature concerning the role of SP and CGRP inducing the angiogenesis process. Using an in vivo sponge model of angiogenesis, Fan TP et al. characterized tachykinin receptors involved in the SP/IL-1α response after subcutaneous implantation of polyether foam [80]. They observed an intense neovascularization, when rats were treated with SP, and even more intense neovascularization when co-treated with both SP and IL-1α. Mapp et al. injected CGRP and its receptor antagonists in knee joints of rats, and they concluded that CGRP stimulates angiogenesis in vivo directly by activating CGRP receptors [81].

The innervation process and sensory neuropeptides also seem to play a remarkable role in the cutaneous wound healing process [82]. Chéret et al. co-cultured injured human skin explants and rat SNs to study the influence of sensory neuropeptides on cutaneous wound healing [45]. The authors observed that SP, CGRP and vasoactive intestinal-related peptide (VIP) regulate MMP2/9 activities, type I and III collagens production, and fibroblast and keratinocyte proliferation.

The upregulation of *Mmp2* by SP was also seen in other cell types such as cardiac fibroblasts [83], human synovial fibroblasts [84], and human pulp cells [85]. In addition, neuropeptides also seem to play an important role in cancer development. For example, Neurokinin-1 receptor (NK-1R) which is the main tachykinin receptor mediating the effects of SP, is overexpressed in several malignant tissues, inducing cell proliferation, migration and neoangiogenesis [86, 87]. Especially, the expression of MMP-2, MMP-9,VEGF-A and VEGF-R1 is upregulated in human KYSE-30 oesophageal squamous cancer cell line when culture medium is supplemented with SP [88]. Taken together, these data are in accordance with our findings, suggesting that innervation and sensory neuropeptides SP and CGRP induce vessel formation and extracellular matrix remodelling in vivo and in vitro in physiological and pathological conditions.

In the present study, when CGRP and SP antagonists were used (Fig. 4), *VegfA*, *Angpt1* and *Col4* expressions were downregulated in the presence of both antagonists used either separately or combined. These results confirm the role of peptidergic and nociceptor SNs in the modulation of the expression of *VegfA*, *Angpt1* and *Col4* in ECs through CGRP and SP. Moreover, even if *Angpt1* and *Col4* gene expression were upregulated only in the presence of SP (Fig. 3), we observed a downregulation in the presence of CGRP antagonist and with both antagonists combined (Fig. 4). These results suggest that even though SP is required for the upregulation of *Angpt1* and *Col4*, this molecule is not the only one responsible for these upregulations. For *Mmp2*, a downregulation was observed only when both agonists were used combined (Fig. 4). Taken together, these findings suggest that SP is required to induce *Mmp2* expression, but since its inhibition is not complete, other molecules might be involved in *Mmp2* upregulation. In fact, for all markers, the amplitude of inhibition by CGRP and SP antagonists was not as important as the upregulation observed in the dose-response experiment. Indeed, it is important to emphasize that when we identified the upregulation of *VegfA* by CGRP, and *Angpt1*, *Col4* and *Mmp2* by SP, these neuropeptides were supplemented directly in ECs culture medium individually (Fig. 3). And when CGRP and SP antagonists were used, SNs and ECs were in indirect co-culture, reflecting a more complex physiological communication between both cell types (Fig. 4). Indeed, the neuropeptides availability and concentrations are not the same in the co-culture model and when ECs alone are treated in culture medium supplemented with the molecules. It is known that SNs secrete other neuropeptides, which could take over or act in synergy when CGRP or SP are inhibited, explaining why the use of SP antagonist does not significantly decrease *Mmp2* gene expression. Another hypothesis is that since the upregulation of *Mmp2* in ECs co-cultured with SNs was around 20-folds, the concentration, exposure time and type of inhibition of CRGP or SP by the antagonists used alone, might not be sufficient to trigger completely the inhibition of this gene expression.

In that way, other subpopulations of SNs into the dorsal root ganglion [55], which secret different neuropeptides other than CGRP and SP, such as neurokinin A, neurokinin B or neurotensin, might be responsible for the *Mmp2* upregulation, in a synergistic or antagonistic manner with SP. There is an ever increasing list of other neuropeptides and bioactive agents released from SNs, some of which are not specific markers from SNs. These include the non-peptide purines and nitric oxide in addition to the neuropeptides galanin, neurotensin, opioids, somatostatin, VIP, and pituitary adenylate cyclase activating peptide (PACAP) [89–91].

## Conclusions

**Fig. 5:**
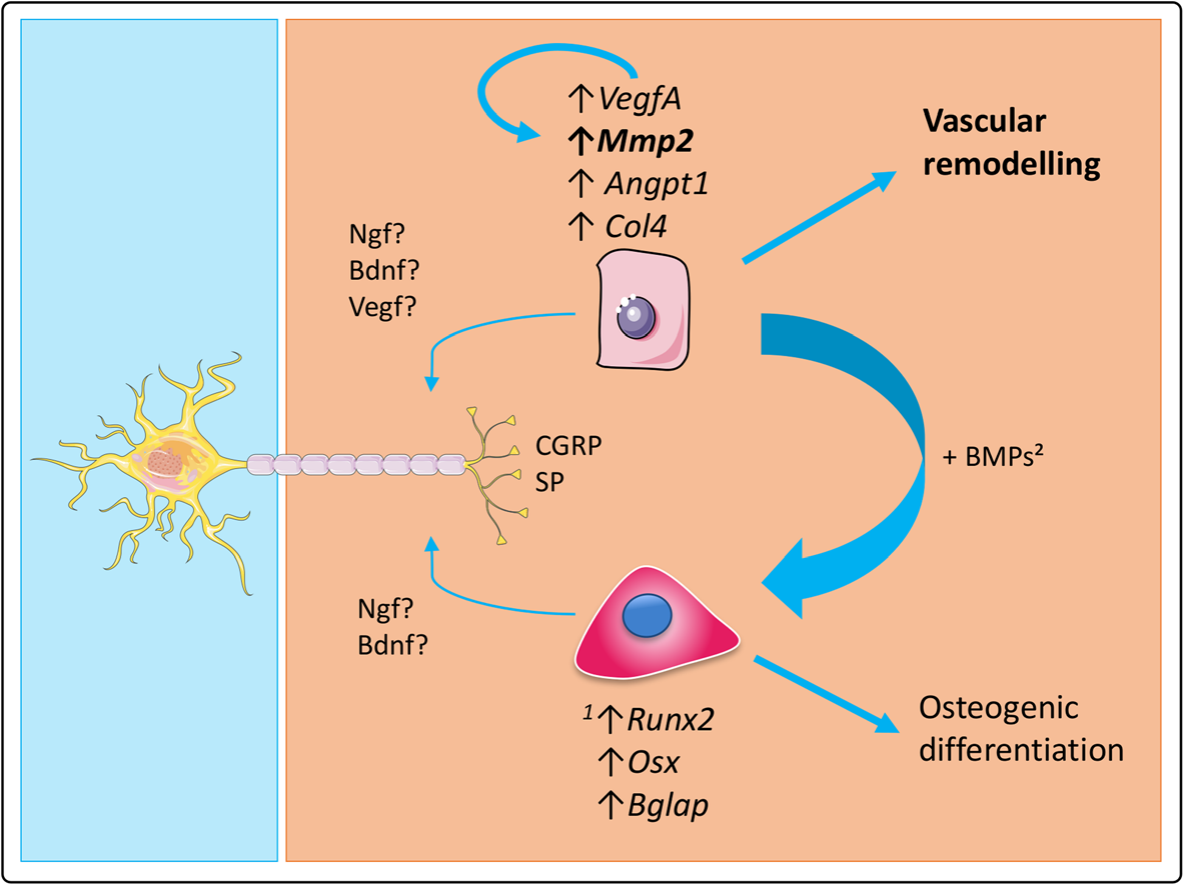
Role of sensory neurons in osteogenic differentiation and vascular remodelling. SNs enhance the osteogenic differentiation of MSCs through the upregulation of genes such as Runx2 (runt-related transcription factor 2), *Sp7* (osterix) or *Bglap* (osteocalcin) [according to Silva et al., 2017 [46]]. Through the secretion of CGRP and SP, SNs also play a role in vascular remodeling by upregulating the expression of some angiogenic markers in ECs. More specifically, they strongly upregulate the expression of *Mmp2* as well as MMP2/MMP9 protein level and enzymatic activity, suggesting a role in angiogenesis through extracellular matrix remodeling. Besides, ECs are known to have an osteoinductive effect on MSC through the release of bone morphogenetic proteins (BMPs) [according to Grellier et al., 2009 [2]], thus the interplay between these three cell types should be considered for the development of bone regeneration strategies

The present study reveals that SNs have a direct effect on ECs functions in view of angiogenesis and ECM remodelling through the upregulation of expression of *VegfA*, *Col4*, *Angpt1* and *Mmp2* (Fig. 2). Peptidergic nociceptors SNs, through the secretion of CGRP and SP, play an important role in this process: CGRP plays a role on *VegfA* upregulation while SP plays a role on *VegfA*, *Angpt1, Col4* and *Mmp2* upregulation (Fig. 3). These neuropeptides seem to be required for gene expression modulation but they are not the only actors involved in this interplay (Fig. 4). The separate evaluation of other subpopulations of SNs could help identify other neuropeptides and mechanisms of action. Our team previously demonstrated that SNs enhance the osteogenic differentiation of MSCs through the upregulation of genes such as *Runx2* (runt-related transcription factor 2), *Sp7* (osterix) or *Bglap* (osteocalcin) [46] (Fig. 5). Moreover, ECs are known to have an osteoinductive effect on MSCs through the release of bone morphogenetic proteins [2], thus the interplay between these three cell types should be considered for the development of future strategies for bone regeneration.

## Data Availability

All data generated or analysed during this study are included in this published article.

## Abbreviations

ANGPT1: Angiopoietin 1
CGRP: Calcitonin gene-related peptide
COL4: Type 4 collagen
DRG: Dorsal root ganglion
EC: Endothelial cell
ECM: Extracellular matrix
MMP: Matrix metalloproteinase
MSC: Mesenchymal stem cell
PECAM1: Platelet endothelial cell adhesion molecule 1
SN: Sensory neuron
SP: Substance P
TEM: Transmission electron microscopy
VEGF: Vascular endothelial growth factor
